# Exploring an AI-First Healthcare System

**DOI:** 10.3390/bioengineering13010112

**Published:** 2026-01-17

**Authors:** Ali Gates, Asif Ali, Scott Conard, Patrick Dunn

**Affiliations:** 1 American Heart Association, 7272 Greenville Avenue, Dallas, TX 7272, USA; 2 Cena Research Institute, 8830 Long Point Rd., Houston, TX 77055, USA; 3 Converging Health, 3890 W. Northwest Hwy, Irving, TX 75039, USA

**Keywords:** artificial intelligence, healthcare delivery, risk stratification, remote monitoring, interoperability

## Abstract

Artificial intelligence (AI) is now embedded across many aspects of healthcare, yet most implementations remain fragmented, task-specific, and layered onto legacy workflows. This paper does not review AI applications in healthcare per se; instead, it examines what an AI-first healthcare system would look like, one in which AI functions as a foundational organizing principle of care delivery rather than an adjunct technology. We synthesize evidence across ambulatory, inpatient, diagnostic, post-acute, and population health settings to assess where AI capabilities are sufficiently mature to support system-level integration and where critical gaps remain. Across domains, the literature demonstrates strong performance for narrowly defined tasks such as imaging interpretation, documentation support, predictive surveillance, and remote monitoring. However, evidence for longitudinal orchestration, cross-setting integration, and sustained impact on outcomes, costs, and equity remains limited. Key barriers include data fragmentation, workflow misalignment, algorithmic bias, insufficient governance, and lack of prospective, multi-site evaluations. We argue that advancing toward AI-first healthcare requires shifting evaluation from accuracy-centric metrics to system-level outcomes, emphasizing human-enabled AI, interoperability, continuous learning, and equity-aware design. Using hypertension management and patient journey exemplars, we illustrate how AI-first systems can enable proactive risk stratification, coordinated intervention, and continuous support across the care continuum. We further outline architectural and governance requirements, including cloud-enabled infrastructure, interoperability, operational machine learning practices, and accountability frameworks—necessary to operationalize AI-first care safely and at scale, subject to prospective validation, regulatory oversight, and post-deployment surveillance. This review contributes a system-level framework for understanding AI-first healthcare, identifies priority research and implementation gaps, and offers practical considerations for clinicians, health systems, researchers, and policymakers. By reframing AI as infrastructure rather than isolated tools, the AI-first approach provides a pathway toward more proactive, coordinated, and equitable healthcare delivery while preserving the central role of human judgment and trust.

## 1. The AI-First Healthcare System: Reimagining Care Delivery

Artificial intelligence (AI) is no longer an emerging concept in healthcare. Over the past decade, advances in machine learning, natural language processing, computer vision, and large language models have led to widespread experimentation and, in some cases, routine deployment of AI systems across clinical domains. AI-enabled tools now support image interpretation, clinical documentation, risk prediction, triage, remote monitoring, and patient engagement. In fields such as radiology, cardiology, and population health analytics, AI systems have demonstrated performance comparable to or exceeding human benchmarks for narrowly defined tasks. As a result, the question facing healthcare is no longer whether AI can be used, but how it should be organized, governed, and integrated to meaningfully improve care delivery.

Despite this progress, most current AI implementations remain fragmented. AI is frequently introduced as a point solution—layered onto existing workflows to address specific inefficiencies or clinical tasks—rather than as a foundational component of healthcare system design. These “AI-assisted” approaches often optimize isolated steps in care (e.g., documentation or image interpretation) without addressing longitudinal coordination, clinician workload redistribution, equity implications, or system-level outcomes. Consequently, many deployments struggle to scale, generalize across settings, or demonstrate sustained impact on patient outcomes, costs, and health equity.

This review argues that realizing the full potential of AI requires a shift from incremental adoption toward an AI-first healthcare paradigm. In an AI-first system, artificial intelligence is not an adjunct technology but a core organizing principle of care delivery—analogous to how “cloud-first” or “mobile-first” strategies reshaped other complex industries. An AI-first approach assumes that data ingestion, risk stratification, workflow orchestration, and feedback loops are continuously enabled by AI, with human clinicians providing oversight, contextual judgment, and relationship-centered care. Importantly, such a system is not defined by automation alone, but by deliberate integration of human expertise, ethical safeguards, and equity-focused design.

While numerous reviews have cataloged AI applications by specialty or task, fewer have examined what healthcare delivery looks like when AI is embedded across the full continuum of care. Existing literature has largely emphasized algorithmic performance metrics, such as accuracy or area under the receiver operating characteristic curve, often in controlled or single-center settings. Less attention has been given to system-level questions: how AI reshapes ambulatory workflows before, during, and after visits; how predictive surveillance alters inpatient decision-making and resource allocation; how remote monitoring and home-based programs extend care beyond clinical walls; and how governance, interoperability, and trust determine real-world impact. Addressing these questions is essential for moving from isolated success stories to sustainable, equitable transformation.

Accordingly, the purpose of this paper is not to provide a comprehensive review of AI technologies in healthcare. Instead, this review synthesizes existing evidence to explore what an AI-first healthcare system would entail, identifying where current AI capabilities are sufficiently mature to support system-level integration and where critical gaps remain. By examining AI applications across ambulatory, inpatient, diagnostic, post-acute, and population health settings, we aim to highlight patterns of success, limitations of current approaches, and design principles necessary for coordinated, longitudinal care.

To guide this analysis, we pose four research questions. First, what distinguishes an AI-first healthcare system from conventional AI-enabled care, and what defining principles characterize this shift? Second, which domains of healthcare demonstrate sufficient evidence to support AI-first workflows, and where do challenges related to validation, generalizability, and equity persist? Third, what technical, organizational, ethical, and policy conditions are required to operationalize AI-first care safely and at scale? Finally, what future research and implementation priorities are needed to advance from pilot AI tools to sustainable, patient-centered AI-first health systems?

By addressing these questions, this review seeks to make several contributions. We provide a critical synthesis of the current AI healthcare literature through a system-level lens, rather than a task-based catalog. We identify gaps in evidence related to integration, equity, and real-world impact. We propose practical frameworks—including implementation readiness criteria and governance considerations—to support clinicians, health systems, and policymakers. Ultimately, we aim to clarify how an AI-first approach can move healthcare toward more proactive, coordinated, and equitable care, while reinforcing the central role of human judgment and trust in clinical decision-making.

## 2. Literature Review

### 2.1. Overview: From Point Solutions to Systems Integration

Foundational reviews consistently characterize artificial intelligence in healthcare as a mature yet uneven field, marked by high performance in narrowly defined tasks alongside limited evidence for system-wide transformation. Large-scale syntheses document robust and reproducible gains in areas such as medical imaging, waveform analysis, and clinical documentation support, while simultaneously highlighting weak generalizability, inconsistent prospective validation, and minimal evaluation of downstream clinical or equity-related outcomes [1,2,3,4,5,6]. Collectively, this literature suggests that while AI has surpassed the experimental phase for specific use cases, it has not yet achieved routine, longitudinal integration into care delivery.

A recurring theme across these reviews is the dominance of accuracy-centric evaluation paradigms. Many studies emphasize algorithmic discrimination, benchmarking against clinician performance, or retrospective validation within single institutions [1,3,6]. However, multiple authors caution that predictive performance alone is insufficient to ensure clinical value. Chen and Asch argue that without alignment to clinical workflows, incentives, and decision contexts, even highly accurate models may fail to improve care or may introduce new risks [7]. Subsequent reviews reinforce this critique, calling for a shift toward implementation-focused evaluations that examine how AI systems influence clinician behavior, patient experience, and outcomes in real-world settings [4,5,8].

More recent consensus and policy-oriented analyses identify governance, safety, and integration—rather than algorithmic innovation—as the primary barriers to scale. These works emphasize that privacy protection, cybersecurity, regulatory oversight, reimbursement alignment, and post-deployment surveillance are foundational requirements for sustainable adoption, not downstream considerations [2,5,9,10,11]. The absence of standardized approaches to model updating, performance drift detection, and accountability has been repeatedly cited as a critical limitation, particularly in high-risk clinical environments where errors can propagate rapidly across systems [10,11].

Equity has emerged as a central concern in system-level analyses of AI in healthcare. While earlier literature treated bias as a secondary or technical issue, more recent syntheses highlight the risk that AI systems trained on non-representative or historically biased data may reinforce disparities in access, diagnosis, and treatment [12,13,14,15]. Reviews focused on responsible and trustworthy AI argue that fairness cannot be addressed solely through post hoc adjustment, but must be embedded across the AI lifecycle—from data curation and validation to deployment and monitoring—especially when AI informs triage, resource allocation, or population health decision-making [10,13,15].

Taken together, these foundational works converge on a shared conclusion: the next phase of AI in healthcare will not be driven by incremental gains in model accuracy, but by the ability to orchestrate AI across workflows, care settings, and time. Rather than evaluating tools in isolation, the literature increasingly calls for system-level frameworks that integrate AI into care pathways, align organizational incentives, and preserve meaningful human oversight. This shift—from “AI-assisted” point solutions to coordinated, AI-enabled healthcare systems—provides the conceptual basis for an AI-first approach and motivates the analysis presented in this review.

### 2.2. Ambulatory Care: Pre-Visit, Triage, Documentation, and Follow-Up

The ambulatory setting has emerged as one of the most active and promising domains for AI deployment, largely because of its high administrative burden, data richness, and longitudinal patient relationships. Environmental scans, qualitative studies, and early implementation reports describe AI systems augmenting nearly every phase of the ambulatory episode, including automated pre-visit planning, symptom intake, triage and routing, real-time documentation support, and post-visit continuity tasks such as referrals, prior authorization, and follow-up communication [16,17,18,19,20]. These applications collectively target inefficiencies that contribute to clinician burnout and fragmented care, positioning ambulatory workflows as a natural entry point for AI-enabled redesign.

Pre-visit and triage applications exemplify the shift from episodic to proactive care. AI-driven intake tools can synthesize structured and unstructured data from electronic health records (EHRs), patient portals, and remote monitoring devices to identify care gaps, stratify risk, and route patients to appropriate levels of care before clinician contact [20,21]. Qualitative evaluations suggest that both patients and clinicians perceive value in these systems when they reduce redundant questioning and clarify visit priorities, although concerns remain regarding transparency, escalation thresholds, and accountability for triage decisions [17,18].

Clinical documentation support—particularly through natural language processing and large language model–based “ambient scribe” systems—represents one of the most rapidly adopted ambulatory AI use cases. Early evidence suggests meaningful reductions in documentation time and cognitive load, with potential downstream benefits for clinician satisfaction and patient engagement [18,21]. However, reviewers consistently caution that these systems require rigorous quality assurance, hallucination mitigation, and clear medico-legal frameworks, particularly when documentation outputs feed directly into billing, clinical decision support, or downstream care coordination [5,18,21].

Direct-to-physician AI reporting in ambulatory diagnostics illustrates a more mature form of AI integration. Continuous ambulatory electrocardiography (ECG) analysis, for example, demonstrates how high-volume signal processing can be algorithmically managed while preserving clinician oversight. Randomized and pragmatic studies show that AI-assisted ECG interpretation can increase detection of clinically actionable arrhythmias with turnaround times compatible with routine outpatient workflows [19]. Importantly, these deployments succeed not merely because of model accuracy, but because outputs are embedded within established clinical pathways and clearly delineate human responsibility for interpretation and follow-up.

Equity considerations are increasingly prominent in ambulatory AI research. Studies focused on language access, health literacy, and patient-clinician communication highlight AI’s potential to reduce barriers to care when tools are co-designed with patients and frontline staff [9,22]. Conversely, reviews caution that poorly designed intake, triage, or chatbot systems may exacerbate disparities if they rely on assumptions about digital access, literacy, or cultural norms [22]. These findings underscore that ambulatory AI systems—particularly those interfacing directly with patients—must be evaluated not only for efficiency and accuracy, but also for accessibility, trust, and differential impact across populations.

Taken together, the ambulatory care literature demonstrates that AI can meaningfully reduce administrative burden and improve workflow efficiency, supporting its role as a cornerstone of an AI-first healthcare system. At the same time, evidence remains uneven with respect to safety, generalizability across EHR platforms, and long-term clinical outcomes. The field would benefit from prospective, multi-site studies that evaluate human-in-the-loop supervision models, equity metrics, and longitudinal effects on care quality. These gaps highlight the distinction between deploying AI tools in ambulatory settings and redesigning ambulatory care around AI-enabled coordination and decision support.

### 2.3. Inpatient and Acute Care: Surveillance, Prediction, and Operational Intelligence

Inpatient and acute care environments represent high-risk, data-dense settings where AI has been increasingly applied to support early detection of clinical deterioration, decision-making under time pressure, and operational coordination. Systematic reviews synthesize a growing body of evidence demonstrating the use of machine learning models for early warning systems, sepsis detection, mortality risk prediction, length-of-stay (LOS) estimation, and bed management optimization [9,23,24]. These applications leverage continuous streams of vital signs, laboratory values, medication data, and clinical notes, positioning AI as a real-time surveillance layer within hospital workflows.

Beyond prediction, narrative and empirical studies describe AI-enabled augmentation of daily inpatient workflows. These include bedside documentation support, automation of repetitive cognitive tasks, and decision support systems that surface salient laboratory trends, imaging findings, and risk signals during clinical rounds [8,18,25,26]. Such tools aim to reduce information overload and cognitive burden in environments characterized by frequent interruptions and high stakes. However, reviews consistently note that the clinical impact of these systems depends less on model sophistication than on how outputs are presented, timed, and aligned with existing team-based decision processes [8,25].

Safety-focused analyses emphasize AI’s potential to reduce adverse events through continuous risk detection and earlier escalation of care. Reviews of AI-enabled early warning and sepsis systems report improved sensitivity compared with traditional rule-based scores, with some studies demonstrating earlier intervention and improved process metrics [8,9,20]. At the same time, authors caution that poorly integrated alerts may contribute to alarm fatigue or erode clinician trust if not paired with clear thresholds, escalation pathways, and human oversight [10,12]. As a result, prospective validation, bias auditing, and integration with rapid response teams are widely cited as prerequisites for safe deployment at scale [10,20].

Operational optimization represents another prominent inpatient AI use case. Predictive models for LOS, discharge readiness, and bed allocation have shown promise in improving throughput and resource utilization, particularly in high-volume hospitals [18,21]. Yet, evidence linking these operational gains to improved patient-centered outcomes remains limited. Reviews note that without alignment to staffing models, incentives, and discharge planning workflows, operational AI tools risk shifting bottlenecks rather than resolving them [27].

Procedural and perioperative applications—including robotic assistance, computer vision–guided surgery, and augmented or virtual reality (AR/VR) overlays—illustrate a more exploratory phase of inpatient AI deployment. Reviews describe promising results in surgical navigation, skill assessment, and intraoperative decision support, but emphasize that most studies remain single-center, device-specific, and limited in their evaluation of clinical outcomes beyond technical feasibility [23,28]. These constraints highlight the challenges of generalizability and integration when AI systems are tightly coupled to proprietary hardware or narrow procedural contexts.

Collectively, the inpatient and acute care literature suggests that AI can meaningfully enhance surveillance, prediction, and operational awareness—capabilities that are central to an AI-first healthcare system. However, the evidence also underscores the risks of fragmented deployment. Without deliberate integration across monitoring, escalation, documentation, and operational workflows, AI systems may improve isolated metrics while failing to deliver system-level gains in safety, efficiency, or equity. Advancing toward AI-first inpatient care will require longitudinal, multi-site evaluations that assess not only predictive accuracy, but also human–AI collaboration, workflow redesign, and downstream clinical outcomes.

### 2.4. Diagnostics and Imaging: From Algorithmic Accuracy to Clinical Integration

Diagnostics and imaging represent some of the most mature and extensively studied applications of artificial intelligence in healthcare. Systematic reviews and meta-analyses consistently report high performance of AI models in detection, classification, and segmentation tasks across radiology modalities including chest radiography, computed tomography (CT), magnetic resonance imaging (MRI), and echocardiography [27,29]. In many cases, AI systems demonstrate accuracy comparable to or exceeding clinician benchmarks for narrowly defined diagnostic tasks, particularly in high-volume, pattern-recognition-intensive workflows. As a result, imaging has often been positioned as a leading indicator of AI’s potential clinical value.

However, contemporary reviews emphasize that technical performance alone does not equate to clinical maturity. Increasing attention has been paid to reproducibility, dataset curation, reporting standards, and external validation across scanners, vendors, and institutions [29,30]. Studies highlight substantial performance variability when models trained in controlled or academic environments are deployed in real-world clinical settings, reinforcing concerns about generalizability and dataset shift. These findings have prompted calls for standardized evaluation frameworks that extend beyond retrospective accuracy metrics to include workflow integration, clinician interaction, and patient outcomes [27,29].

The emergence of generative AI and large language models in diagnostic reasoning has further sharpened these concerns. Scoping reviews and empirical studies evaluating generative AI on complex diagnostic case series report heterogeneous performance, with models demonstrating strengths in pattern synthesis and summarization but weaknesses in clinical reasoning consistency, error calibration, and transparency [31,32]. These results underscore the necessity of guardrails, expert oversight, and clear delineation of responsibility when generative models are applied to diagnostic contexts, particularly where outputs may influence downstream clinical decisions.

Ambulatory electrocardiography (ECG) analytics illustrate a more advanced stage of diagnostic AI integration. Continuous ECG monitoring systems combine high-volume signal processing with clinician-facing reports that fit within established diagnostic and follow-up workflows. Randomized and pragmatic trials demonstrate improved detection of clinically actionable arrhythmias, such as atrial fibrillation, with turnaround times compatible with routine outpatient care [19,33]. Importantly, these deployments succeed not only due to algorithmic accuracy, but because they embed AI outputs within defined clinical pathways, preserving physician interpretation, accountability, and patient communication.

Across diagnostic domains, the literature increasingly reflects a shift away from isolated performance metrics—such as area under the receiver operating characteristic curve (AUROC)—toward broader questions of clinical impact, human factors, and equity. Reviews now emphasize the importance of evaluating how AI tools affect diagnostic confidence, workflow efficiency, inter-reader variability, and access to care, particularly across diverse patient populations and imaging environments [29,30,31,32]. Equity-focused analyses caution that differential performance across subgroups or imaging infrastructure may exacerbate disparities if not systematically measured and addressed [31,34].

In summary, diagnostic AI has reached a level of technical sophistication that supports its inclusion in an AI-first healthcare system. Yet, the literature makes clear that diagnostic excellence alone is insufficient. Realizing system-level value requires integration across data pipelines, clinical workflows, and governance structures, alongside rigorous evaluation of safety, equity, and longitudinal outcomes. Imaging thus serves both as a model of AI’s potential and a cautionary example of the limitations of task-centric adoption absent system-level design.

### 2.5. Population Health and Public Health: Risk Stratification, Learning Systems, and Governance at Scale

At the population health and public health level, artificial intelligence has been widely proposed as a mechanism to shift healthcare from reactive treatment toward proactive prevention and continuous learning. Reviews and policy-oriented commentaries describe AI applications in risk stratification, disease surveillance, outbreak detection, screening optimization, and learning health systems, emphasizing their potential to target preventive services, personalize outreach, and allocate resources more effectively [2,7,10,12,14,34]. These applications leverage large, heterogeneous datasets—including electronic health records, claims data, registries, social determinants of health, and, increasingly, non-traditional data sources—to inform decisions at the level of populations rather than individual encounters.

A consistent theme across this literature is that governance, rather than model performance, is the primary limiting factor for AI deployment at scale. When AI outputs are used to prioritize patients, communities, or interventions, questions of dataset provenance, representativeness, and drift monitoring become central to ethical and operational viability [10,35]. Reviews emphasize that population-level AI systems must be continuously audited not only for predictive accuracy, but also for differential performance across demographic and socioeconomic subgroups, particularly when outputs influence access to preventive services or resource allocation [12,15,34].

Several high-profile analyses illustrate the risks of insufficient governance. Studies examining population health algorithms used for care management and resource prioritization have demonstrated that models optimized for cost or utilization proxies may systematically disadvantage historically marginalized populations, even when race or ethnicity are not explicitly included as inputs [27,34]. These findings have catalyzed calls for explicit alignment between AI objectives and equity metrics, as well as greater transparency regarding model assumptions, intended use, and limitations [10,12,35].

Despite strong methodological interest, implementation evidence at the population and public health level remains limited. While numerous studies propose advanced risk prediction models or surveillance architectures, relatively few trace the full arc from prediction to intervention to measurable changes in outcomes, costs, or disparities in real-world settings [7,14,36]. Reviews of learning health systems note that feedback loops—where model outputs inform action and outcomes are fed back into model refinement—are often conceptual rather than operational, constrained by fragmented data systems, unclear accountability, and misaligned incentives [7,35].

From an AI-first perspective, population health represents a critical integration layer that connects individual-level predictions with system-level decision-making. However, the literature makes clear that achieving this vision requires more than sophisticated analytics. Sustainable population-level AI systems demand robust data governance, explicit equity safeguards, interdisciplinary oversight, and alignment with public health infrastructure and policy goals. Advancing this domain will require prospective evaluations that assess not only predictive performance, but also implementation fidelity, community impact, and long-term value—ensuring that AI-first population health initiatives contribute to more equitable and effective care at scale.

### 2.6. Post-Acute, Home-Based, and Long-Term Care: Extending AI-Enabled Care Beyond Clinical Walls

Post-acute, home-based, and long-term care settings represent a critical frontier for AI-enabled healthcare, as they extend care beyond episodic encounters and into patients’ daily lives. Comparative analyses, qualitative studies, and early implementation reports describe AI-supported remote monitoring, triage, and team-based coordination as mechanisms to identify clinical deterioration earlier, reduce avoidable readmissions, and surface safety risks that might otherwise go undetected between visits [15,37,38,39]. These settings are particularly well suited to AI-first approaches because they rely on continuous data streams, longitudinal risk assessment, and coordination across multidisciplinary teams.

Remote patient monitoring (RPM) systems are among the most widely studied applications in this domain. AI-enabled RPM platforms integrate physiologic data—such as blood pressure, heart rate, glucose levels, and activity metrics—with symptom reports and contextual information to support dynamic risk stratification and escalation [15,38]. Reviews suggest that when paired with defined response protocols and clinical oversight, these systems can improve detection of decompensation and enhance continuity of care, particularly for chronic cardiovascular and metabolic conditions. However, evidence also highlights substantial variability in implementation, with outcomes dependent on staffing models, workflow integration, and patient engagement strategies rather than algorithmic performance alone [37,38].

Hypertension-focused primers and specialty-specific frameworks provide an instructive example of how AI can be operationalized in post-acute and home-based care. These works emphasize the importance of standardized terminology, clearly defined care pathways, and explicit expectations for model use when integrating AI-enabled monitoring into routine clinical practice [37]. By codifying how data are collected, interpreted, and acted upon, such frameworks reduce ambiguity for clinicians and patients alike, supporting safer and more consistent deployment. Importantly, these efforts illustrate how AI-first design at the program level can align technology, workflows, and clinical goals rather than introducing isolated tools.

Long-term care and home health settings also surface distinct equity and accessibility considerations. Reviews of AI in these contexts underscore challenges related to digital literacy, device access, caregiver burden, and trust—factors that can limit the effectiveness of AI-enabled interventions if not addressed proactively [15,39,40]. At the same time, qualitative studies suggest that when AI tools are designed with patients, caregivers, and frontline staff, they may enhance communication, support shared decision-making, and reduce unnecessary escalation of care [39]. These findings reinforce the need for human-centered and equity-aware design in AI-first home-based care models.

Across post-acute and long-term care settings, the strongest evidence to date supports feasibility, acceptability, and process improvements rather than definitive effects on clinical outcomes or costs. Few studies report multi-site, prospective evaluations that trace the full pathway from AI-enabled monitoring to intervention, outcome change, and economic impact [37,38,40]. As a result, the literature calls for more rigorous implementation studies that assess scalability, sustainability, and value in real-world health systems.

From an AI-first perspective, post-acute and home-based care illustrate both the promise and the challenges of continuous, data-driven healthcare. These settings demonstrate how AI can enable longitudinal risk management and coordinated response, but they also expose the limitations of deploying technology without aligned workflows, reimbursement models, and governance structures. Advancing AI-first care beyond the hospital will require robust outcome evaluation, explicit equity safeguards, and sustained investment in the human infrastructure that translates prediction into action.

### 2.7. Architecture and Systems: The Foundation of an AI-First Healthcare Ecosystem

An AI-first healthcare system depends not only on advanced algorithms, but on a robust architectural foundation capable of integrating data, workflows, and human oversight across the continuum of care. Unlike traditional health information technology—often designed for documentation or billing—AI-first architecture must support continuous data ingestion, real-time analytics, bidirectional clinical workflows, and longitudinal learning. As such, architecture functions as a core determinant of whether AI operates as an isolated tool or as a system-level capability embedded in care delivery.

Most contemporary analyses converge on the need for cloud-based and hybrid architectures to support AI-first healthcare. Cloud infrastructure enables the scalability, computational elasticity, and model deployment pipelines required for large-scale machine learning, while hybrid approaches allow sensitive data processing to remain within institutional or jurisdictional boundaries when needed [15,41]. However, infrastructure alone is insufficient. Secure data access, encryption, role-based permissions, and auditability are essential design requirements, particularly when AI systems process high-frequency physiologic data or sensitive clinical narratives.

Interoperability is a defining prerequisite for AI-first systems. AI-enabled care depends on the seamless exchange of structured and unstructured data across electronic health records, imaging systems, laboratory platforms, remote monitoring devices, and patient-facing tools. Reviews consistently identify fragmented data standards and limited semantic interoperability as major barriers to effective AI deployment [11]. Without standardized data models and reliable data pipelines, AI systems are unable to support longitudinal risk stratification, cross-setting coordination, or learning health system feedback loops. In practice, AI-first interoperability depends on adoption of established standards, including HL7 FHIR for structured clinical data exchange, DICOM for imaging workflows, and common data models such as OMOP/CDM to support analytics, population health, and cross-site evaluation of AI models [27].

Within this architectural foundation, AI-first care relies on orchestrated system components rather than standalone applications. These include pre-engagement systems that synthesize patient data prior to encounters, identifying risks, care gaps, and priorities; in-encounter systems that support documentation, decision-making, and information synthesis; and post-engagement systems that monitor outcomes, adherence, and recovery while enabling timely escalation when needed [11]. Importantly, these components must be designed to work together, with clear handoffs and shared data context, rather than operating as parallel or competing tools.

Ambient and conversational AI systems exemplify both the promise and the challenges of AI-first architecture. When integrated effectively, such systems can reduce documentation burden and surface clinically relevant information in real time. However, their value depends on accurate capture, transparent processing, and alignment with clinical accountability. Reviews emphasize that outputs must remain interpretable, editable, and attributable to clinicians, reinforcing the principle of human-enabled AI rather than autonomous decision-making [10,11].

Underlying all architectural layers are algorithmic pipelines that require continuous governance. AI-first systems must support version control, performance monitoring, bias auditing, and model updating over time. Static deployment models are insufficient in clinical environments where population characteristics, clinical practices, and data quality evolve. As a result, recent literature stresses the importance of operational machine learning practices (MLOps) and post-deployment surveillance as core architectural capabilities, not optional add-ons [17,18]. Beyond conventional health IT security concerns, AI-first healthcare systems introduce additional model-level vulnerabilities, including data poisoning during training, model inversion or membership inference attacks that can expose sensitive patient information, and adversarial inputs designed to manipulate model outputs, necessitating security-aware design, continuous monitoring, and post-deployment risk management tailored specifically to machine learning systems [24].

Despite architectural advances, significant technical and organizational challenges persist. Data quality and fragmentation remain pervasive, limiting model reliability and generalizability. Algorithmic bias continues to pose risks when training data reflect historical inequities or incomplete population representation. Explainability in AI-first healthcare systems encompasses both inherently interpretable models and post hoc explanation techniques, each with distinct trade-offs. Inherently interpretable approaches, such as logistic regression, generalized additive models, and decision trees—offer transparency by design and may be preferable in high-stakes or regulatory-sensitive clinical contexts. In contrast, complex models such as deep neural networks often rely on post hoc explanation methods (e.g., SHAP or LIME) to approximate feature influence after model training; while useful for auditing and sense-making, these techniques provide local or surrogate explanations and may not faithfully represent underlying model behavior, particularly in clinical settings where trust and accountability are paramount [14]. AI-first system design must therefore align model choice and explanation strategy with clinical risk, accountability requirements, and user trust, rather than treating explainability as a uniform or purely technical feature. Finally, integration with legacy health IT systems and alignment with clinical workflows often determine success or failure more than algorithmic performance itself [10,12,14,42].

Addressing these challenges requires coordinated action across technology development, clinical leadership, and policy. Standardized data models, shared governance frameworks, and interdisciplinary oversight structures are increasingly viewed as prerequisites for AI-first healthcare. Equally important is investment in the human infrastructure—training clinicians to interpret AI outputs, defining accountability structures, and ensuring that systems are designed around real-world clinical practice rather than idealized workflows.

In summary, architecture and systems design form the backbone of an AI-first healthcare ecosystem. When thoughtfully implemented, they enable continuous learning, coordinated care, and responsible human–AI collaboration. When neglected, they constrain AI to isolated use cases with limited impact. Advancing toward AI-first healthcare therefore requires treating architecture not as a technical afterthought, but as a strategic enabler of safe, equitable, and sustainable care delivery.

## 3. Conclusions: Embracing the Future of AI in Healthcare

Artificial intelligence has reached a level of technical maturity that makes its widespread presence in healthcare inevitable. Yet, as this review has shown, the transformative potential of AI does not lie in the proliferation of isolated tools, but in a deliberate shift toward an AI-first healthcare system-one in which AI serves as a foundational infrastructure for care delivery rather than an adjunct to existing workflows. The distinction between AI-assisted care and AI-first care is not semantic; it reflects fundamentally different assumptions about how clinical work is organized, how decisions are supported, and how care is coordinated over time.

As summarized across care domains in Table 1, the literature reviewed here demonstrates that AI has achieved meaningful success in narrowly defined tasks, particularly in diagnostics, documentation, predictive analytics, and remote monitoring. In ambulatory settings, AI-enabled pre-visit planning, triage, and documentation have shown promise in reducing administrative burden and improving continuity. In inpatient and acute care, predictive surveillance and operational optimization suggest opportunities for earlier intervention and more efficient resource use. Post-acute and home-based programs illustrate how AI can extend care beyond traditional settings through continuous monitoring and adaptive support. However, across these domains, most implementations remain fragmented and additive, with limited evidence of longitudinal orchestration, cross-setting integration, or sustained impact on outcomes and equity.

A central finding of this review is that the primary barriers to AI-first healthcare are no longer purely technical. Instead, challenges related to data quality and interoperability, algorithmic bias, explainability, workflow integration, and governance consistently limit scale and generalizability. These constraints underscore the importance of human-enabled AI, in which clinicians remain accountable for decisions, interpret model outputs within clinical context, and maintain the relational aspects of care that AI cannot replicate. An AI-first system, therefore, does not diminish the clinician’s role; it reshapes it, shifting effort away from routine cognitive and administrative tasks toward judgment, communication, and shared decision-making.

This review also highlights persistent gaps in the current evidence base. Few studies evaluate AI systems prospectively across multiple sites or diverse populations, and even fewer assess downstream effects on health disparities, cost, or patient experience. Equity considerations are frequently acknowledged but inconsistently operationalized, with limited use of standardized bias audits or health literacy–adapted outputs. Similarly, governance frameworks for monitoring model drift, updating algorithms, and assigning accountability remain underdeveloped. Addressing these gaps is essential if AI-first care is to advance beyond high-resource settings and deliver on its promise of more equitable healthcare.

Looking forward, the transition to AI-first healthcare will require coordinated advances across research, practice, and policy. Future studies should prioritize system-level evaluations that measure clinical outcomes, operational performance, and equity impacts in real-world settings. Health systems must invest in interoperable infrastructure, human-centered design, and workforce training that supports effective human–AI collaboration. Policymakers and regulators play a critical role in establishing standards for safety, transparency, and post-deployment surveillance that reflect the dynamic nature of AI systems. Importantly, reimbursement and value-based care models must evolve to recognize the longitudinal benefits of AI-enabled prevention, monitoring, and coordination. Where feasible, future AI-first healthcare research should prioritize sharing of code, model documentation, and evaluation protocols, or clearly justify restrictions, to support reproducibility.

In conclusion, an AI-first healthcare system represents a shift in design philosophy rather than a simple accumulation of new technologies. As illustrated across domains in Table 1, realizing this vision will require moving beyond isolated successes to integrated systems that learn continuously, adapt responsibly, and ultimately enhance—not replace—the human foundations of medical care.

## Figures and Tables

**Table 1:** Summary of Evidence Supporting an AI-First Healthcare System Across Care Domains.

Section	Care Domain	Primary AI Capabilities	System-Level Contribution	Key Evidence (Ref.)
1	From Point Solutions to Systems Integration	Predictive modeling, NLP, ML pipelines	Establishes need to move from task-specific AI toward longitudinal, integrated care systems; emphasizes governance, equity, and workflow alignment	[1,2,3,4,5,6,7,9,10,11,12,13,14,15]
2	Ambulatory Care	Pre-visit planning, triage, documentation automation, ECG analytics	Enables proactive care, reduces administrative burden, supports continuity before and after visits	[16,17,18,19,20,21,22]
3	Inpatient & Acute Care	Early warning systems, sepsis prediction, operational intelligence	Enhances real-time surveillance, patient safety, and resource optimization while requiring human-in-the-loop oversight	[8,9,10,20,25,26,27]
4	Diagnostics & Imaging	Computer vision, generative AI, signal processing	Demonstrates high technical maturity; highlights need for workflow integration, accountability, and equity-aware deployment	[27,28,29,30,31,32,33]
5	Population & Public Health	Risk stratification, learning health systems, surveillance analytics	Supports proactive prevention and resource allocation; underscores governance and equity as primary constraints	[2,7,10,12,14,34,35,36]
6	Post-Acute, Home-Based & Long-Term Care	Remote monitoring, adaptive risk escalation, care coordination	Extends AI-enabled care beyond clinical walls; enables longitudinal monitoring and early intervention	[15,37,38,39,40]
7	Architecture & Systems	Cloud/hybrid infrastructure, interoperability, MLOps, governance	Provides foundational infrastructure for AI-first care, enabling scalability, continuous learning, and responsible deployment	[10,11,14,17,18,24,41,42]

## Data Availability

No new data were required or available.
